# Seizures in Children with SARS-CoV-2 Infection: Epidemiological, Clinical and Neurophysiological Characterization

**DOI:** 10.3390/children9121923

**Published:** 2022-12-08

**Authors:** Antonia Pascarella, Marco Maglione, Selvaggia Lenta, Elisa Sciorio, Raffaele Mancusi, Celeste Tucci, Francesca Angrisani, Sabrina Acierno, Camilla Calì, Vincenzo Tipo, Antonietta Giannattasio

**Affiliations:** 1 Pediatric Emergency Department, Santobono-Pausilipon Children’s Hospital, 80121 Naples, Italy; 2 Department of Neuroscience, Pediatric Neurology, Santobono-Pausilipon Children’s Hospital, 80121 Naples, Italy; 3 Childhood Cancer Registry of Campania, Santobono-Pausilipon Children’s Hospital, 80121 Naples, Italy

**Keywords:** COVID-19, children, neurological manifestations, seizures

## Abstract

Severe acute respiratory syndrome coronavirus 2 (SARS-CoV-2) may present with a wide variety of symptoms, including neurological manifestations. We investigated clinical, demographic, laboratory, neurophysiological and imaging characteristics of SARS-CoV-2-positive children with seizures and analyzed differences between children admitted during the periods with prevalent circulation of the Alpha/Delta and Omicron variants, respectively. Patients’ characteristics were analyzed according to the presence or absence of seizures and then according to the SARS-CoV-2 variants. Five-hundred and four SARS-CoV-2-positive patients were included: 93 (18.4%) with seizures and 411 (81.6%) without. Patients with seizures were older, had more commonly an underlying epilepsy and had more frequently altered C-reactive protein than those without seizures. Electroencephalography was abnormal in 5/38 cases. According to the SARS-CoV-2 variant, seizures were recorded in 4.7% of the total number of hospitalized patients during the Alpha/Delta period, and in 16.9% of patients admitted during the Omicron period. During the Alpha/Delta variants, seizures were more commonly observed in patients with epilepsy compared to those observed during the Omicron period. Our findings suggest that although SARS-CoV-2 may potentially trigger seizures, they are generally not severe and do not require intensive care admission.

## 1. Introduction

Severe acute respiratory syndrome coronavirus-2 (SARS-CoV-2) is a mild disease in most pediatric patients [1,2]. Symptomatic children generally present only minor respiratory or gastrointestinal symptoms. The risk of hospitalization and of a severe clinical course is mainly related to the development of the post-infectious multi-system inflammatory syndrome (MIS-C) [3,4]. During the pandemic, many variant strains have emerged, and the clinical spectrum of coronavirus disease 2019 (COVID-19) changed according to the SARS-CoV-2 variants. Particularly, the Delta variant has been associated with a more severe disease [5]. Since January 2022, the Omicron variant has been predominant worldwide [6]. Despite being more contagious, this variant seems to be responsible for less severe clinical manifestations than previous strains [7,8,9]. A relevant aspect of pediatric COVID-19 is the possible involvement of the central or peripheral nervous system at various degrees of severity [10]. Most pediatric studies have focused on respiratory symptoms and MIS-C, whereas the spectrum of neurological involvement in children hospitalized with acute COVID-19 has been less investigated. The studies performed during the circulation of the Delta variant indicated that seizures were not a common neurological manifestation of COVID-19 in children [11]. Conversely, more recent investigations reported seizures as an early sign of acute SARS-CoV-2 infection in children during the spread of the Omicron variant [12]. Data on the characterization of seizures in children with COVID-19 are scarce. The aim of this study was to describe clinical, demographic, laboratory, neurophysiological and imaging characteristics of hospitalized children with seizures and SARS-CoV-2 positivity. As a secondary aim, we analyzed the differences between children admitted during the periods with prevalent circulation of the Alpha/Delta and Omicron variants, respectively.

## 2. Materials and Methods

The present retrospective study was performed at the “Santobono-Pausilipon Children’s Hospital”, one of the largest Italian Pediatric Centers in charge of the management of children with SARS-CoV-2 infection. The period of enrollment ranged from September 2020 to August 2022. Patients were considered eligible if they were hospitalized for acute illness for more than 48 h, were younger than 14 years and had a positive SARS-CoV-2 test result (reverse transcriptase polymerase chain reaction on naso-pharyngeal swab). All SARS-CoV-2-positive children admitted with seizures were included. Patients with neurological symptoms other than seizures and non-specific neurological symptoms as headache and myalgia were not included in the study, as well as patients with a diagnosis of MIS-C. Demographic, clinical data, laboratory findings and clinical outcome were analyzed. Furthermore, neurophysiological tests and neuroradiological information, when available, were collected.

Patients’ characteristics were first analyzed according to the presence or absence of seizures as clinical manifestation of COVID-19. To analyze differences according to the SARS-CoV-2 variants, patients were then divided in two groups depending on the time of hospital admission (before and after January 2022). These time frames were established considering that in European countries, including Italy, the Delta variant was dominant until 5 December 2021, while the Omicron variant became dominant since 3 January 2022, with an intermediate period in which prevalence transitioned from Delta to Omicron [6].

### Statistical Analysis

Statistical analysis was performed using StataCorp LLC Stata 17.0 (College Station, TX, USA). Categorical variables are reported as number and percentage, while continuous variables are shown as median and interquartile ranges (laboratory results) or as mean and standard deviation (SD). Demographic, clinical and laboratory features were compared between patients with and without neurological manifestations and between patients observed before and after January 2022. Categorical variables were evaluated with Chi-square test or Fisher’s exact test (when values in any of the cells of a contingency table were below 5), while Wilcoxon’s rank sum test was used for continuous variables. A two tailed *p*-value < 0.05 was considered statistically significant.

## 3. Results

### 3.1. Population

A total of 795 patients with acute SARS-CoV-2 infection were hospitalized during the study period (339 patients between September 2020 and December 2021 and 456 between January 2022 and August 2022). Of them, 504 patients were included in the analysis; 93 (18.4%) positive children (age range, 2.5 months–12.7 years) presented seizures at onset or during COVID-19. The remaining 411 (81.6%) children (age range, 20 days–13 years), admitted with a diagnosis of COVID-19 and absence of neurological manifestations, served as the control group. Overall, 100 (19.8%) patients had an underlying chronic disease.

### 3.2. Patients’ Characteristics

Comparison between patients with and without SARS-CoV-2 infection-related seizures are presented in Table 1. Patients with seizures were older than the group without seizures and had more commonly an underlying disease (mainly an epileptic disorder). As for inflammatory parameters, patients with seizures presented more frequently a pathological value of C-reactive protein (CRP) compared to the group without seizures (*p* < 0.05). Seizures (with or without fever) were the only presenting complaint of SARS-CoV-2 infection in 77/93 (82.8%) patients, while 16 (17.2%) patients presented seizures during hospital observation. A new-onset seizure manifested in 65 (69.9%) patients, while a previous history of febrile seizures and of epilepsy was documented in 10 (10.7%) and in 18 (19.3%) patients, respectively.

As for the type of seizure, 59 (63.4%) patients presented a febrile seizure, while 34 (36.6%) children were admitted with seizures in the absence of fever. Seventeen (28.8%) patients with febrile seizures and six (17.6%) patients with afebrile seizures were less than 1 year old (*p* > 0.05), whereas thirteen (22%) and eighteen (52.9%) patients were older than 5 years, respectively (*p* > 0.05). Furthermore, 7/59 (11.9%) patients with febrile seizures had an underlying diagnosis of epilepsy versus 11/34 (18.6%) patients from the afebrile seizures group (*p* = 0.03).

Generalized, tonic-clonic seizures occurred in 91 (97.8%) patients while partial seizures were observed in two cases only. Three (3.2%) patients presented with a status epilepticus; none had a history of prior status epilepticus, but they all had preexistent neurological conditions. Particularly, a 10-year-old female had a severe neurological disability and epilepsy on medical treatment; a 2-year-old female had a previous diagnosis of genetic epilepsy, and the third patient (a 7-year-old male) had a previous diagnosis of autism spectrum disorder without prior history of seizures. In 66/93 (71%) cases a single seizure was recorded, while 27 (29%) patients had more than one seizure (range, 2–6 episodes) during the hospital stay.

Then, we characterized seizures in 75 patients without a previous diagnosis of epilepsy (23 patients with afebrile seizures and 52 patients with febrile seizures). Seizures were generalized in all cases. Nine/twenty-three (39.1%) patients with afebrile seizures and ten/fifty-two (19.2%) patients with febrile seizures had more than one episode (range 2–6) (*p* > 0.05). As for age, a significantly lower percentage (n = 8/23, 34.8%) of patients with afebrile seizures had ≤2 years compared to the non-epileptic group with febrile seizure (n = 36/52, 69.2%) (*p* = 0.005).

As for neurophysiological findings, 38/93 (40.9%) patients underwent electroencephalography (EEG) during the hospital stay. Findings were normal in 33/38 (86.8%) cases. An abnormal interictal EEG was detected in five (13.2%) cases. In one case we found sporadic epileptiform activity in the occipital lobe. In three cases we found focal non-epileptiform abnormalities of the occipital lobe. Subcontinuous bitemporal sharp waves were found in one patient.

Neuroimaging studies (head computed tomography, CT, in all cases) were performed in 23/93 (24.7%) patients, and they were unremarkable (except for previously known findings) in all cases. No patient underwent brain magnetic resonance imaging (MRI) or lumbar puncture for cerebrospinal fluid (CSF) examination.

Twelve (12.9%) out of ninety-three patients received benzodiazepines. Seven (7.5%) patients required a continuous intravenous midazolam infusion (in three cases because of the development of a status epilepticus and in four cases because of refractory generalized seizures. Among the 18 patients already on antiepileptic medications, in 7 cases a modulation of the ongoing therapy (increase in dosage or introduction of a new antiepileptic drug) was required. Overall, in 5/93 (5.4%) patients an ex-novo antiepileptic therapy was started. The chosen medications were carbamazepine and valproate in two and two cases, respectively, and phenobarbital in one patient. Of them, only one patient had an underlying disease (autism spectrum disorder). In four of these five patients, the EEG was pathological. The remaining child who started an antiepileptic therapy was a 70-day-old male observed during the period of the Omicron variant circulation, who presented three subsequent generalized febrile seizures requiring intravenous phenobarbital, switched to oral therapy at hospital discharge.

No patient had a severe SARS-CoV-2-related respiratory involvement. Furthermore, no patient had neurological sequelae or died.

### 3.3. Characteristics of Patients with Seizures According to SARS-CoV-2 Variants

SARS-CoV-2 infected patients with seizures were divided in two groups according to the time of admission. Sixteen patients were admitted before January 2022 (Group 1) while the remaining seventy-seven were observed after January and represented the Omicron group (Group 2). Group 1 represented the 4.7% of the total number of hospitalized patients during the Alpha/Delta period (*n* = 339), while Group 2 represented the 16.9% of patients admitted during the Omicron variants period (*n* = 456) (*p* < 0.0001).

Table 2 summarizes the differences between the two groups. Patients from Group 2 were younger than those from Group 1. During the Alpha/Delta variants, seizures were more commonly observed in patients with a chronic epileptic condition and were more often afebrile seizures compared to patients observed during the Omicron variant period. In the group of 54 patients with febrile seizures admitted during the Omicron period, 14 (25.9%) were less than 1 year old and 12 (22.2%) were older than 5 years.

Most patients (n = 66, 71%) had one seizure, while multiple episodes (range, 2–6) occurred in 27/93 (29%) cases, without differences between the two groups.

EEG abnormalities were detected in 3/7 (43%) and 2/31 (6.4%) children of Group 1 and Group 2, respectively (*p* = 0.04). A total of 6 out of 16 (37.5%) patients were already on antiepileptic therapy in Group 1, versus 12/77 (15.6%) in Group 2 (*p* = 0.04).

## 4. Discussion

To the best of our knowledge, this is the first study describing clinical, demographic, laboratory and neurophysiological findings in a large cohort of children with seizures concomitant to acute SARS-CoV-2 infection. Neurological involvement during COVID-19 is frequent in adult patients, with headache and anosmia representing the most common symptoms reported [13]. While severe cerebrovascular events are widely reported in SARS-CoV-2-infected adults [13], headache, seizures and peripheral nervous system involvement were more commonly described in pediatric patients, with a 1% of severe brain involvement [14,15,16]. Our findings suggest that seizures may be the initial manifestation of SARS-CoV-2 infection in children, also in the absence of a prior history of neurological disorders. Several large studies reporting neurological manifestations as part of the clinical picture of pediatric COVID-19 are not specifically focused on seizures [1,17]. On the other hand, other studies focused on neurological manifestations included patients with MIS-C [12,14,18]. Furthermore, the prevalence of seizures varies according to the study period. The description of seizures in children with COVID-19 was anecdotal at the beginning of the pandemic [11]. A study including children observed before the Omicron variant spread reported seizures in 1.6% of 243 patients [19]. Cadet et al. recently reported a prevalence of febrile seizures of 0.5% in a multicenter study from US [11]. Again, this study included patients observed until April 2021. Kurd et al. characterized clinical and demographic data of 11/175 (6%) SARS-CoV-2-positive children presenting at the Emergency Department because of seizures between March and December 2020 [20]. On the other hand, studies from South Africa conducted during the first wave (December 2021) of the Omicron variant revealed that seizures were observed in about 20% of hospitalized children with COVID-19 [21]. These data are in line with an Italian preliminary study that reported a rate of seizures of 21% during the first month of Omicron predominance [12]. Overall, we found a prevalence of about 18% of seizures in our study population. Nevertheless, the prevalence deeply changed when we considered the rate of seizures in relationship to the two study periods, with a significant higher rate of SARS-CoV-2-positive children experiencing seizures during the Omicron variant period compared to the previous epidemic waves. Furthermore, we well characterized clinical, laboratory and neurophysiological findings in our patients. As for the age of patients, we observed cases with febrile seizures outside the common expected age range (1–5 years). Specifically, 18/93 (19%) patients were older than 5 years. This implies that SARS-CoV-2 (mainly the Omicron variant) may have a more pronounced neurotropism compared to other common viruses in children. Incidence and age distribution of febrile seizures during the Omicron spread resembles what is described for Influenza infection. It is well known that Influenza A and B, together with coronaviruses, parainfluenza and enteroviruses, cause more Emergency Department visits because of febrile seizures compared to other respiratory viruses [22]. In a recent study, Influenza was detected as the second most frequently detected pathogen in children with febrile seizures [23]. Furthermore, the mean age of patients with febrile seizures due to single Influenza infection was higher (42.4 ± 26.1) than age of those positive for Human Herpes Virus 6 (18.1 ± 4.8), Adenovirus (27.5 ± 12.5) or Rhinovirus (19.6 ± 7.6) [23].

It is unclear if the high incidence of seizures with the Omicron variant is due to the neurotropism of the virus or to a higher number of children experiencing fever or higher fever spikes during Omicron infection compared to previous variants. It has been reported that fever is a clinical manifestation in 84% of Omicron-infected children, with a mean fever spike of 38.9 ± 0.6 °C [24]. The febrile response in patients with febrile seizures could also reflect an altered function of the cytokine network, with IL-1 and IL-6 being the most likely involved mediators [25]. On the other hand, it is known that the SARS-CoV-2 virus induces a systemic inflammatory response responsible of severe clinical spectrum at least in adults. This mechanism could contribute to an amplification of the cytokine release underlying the pathogenesis of febrile seizures [26].

Seizures are also among the most common neurological manifestations related to Rotavirus infection in children, occurring in approximately 1–8% of patients with Rotavirus gastroenteritis. This clinical condition is seen in previously healthy children aged 1 month to 6 years (peaking in 1–2-year-olds) and present with afebrile (or febrile) seizures that develop 1–6 days after gastroenteritis onset [27]. However, our data suggest that, compared to Rotavirus, seizures during SARS-CoV-2 infection seem to affect older children. Nevertheless, similarly to Rotavirus, SARS-CoV-2-related seizures presented as repeated episodes in more than one third of cases in our cohort.

It has been reported that children with SARS-CoV-2 infection or MIS-C with preexisting conditions are at increased risk to develop neurological manifestations [28]. In our study, children with epilepsy had higher risk to experience seizures during COVID-19 compared to patients without underlying neurological diseases (54% versus 9%). The impact of epilepsy on the risk to develop seizures was significantly reduced during the Omicron wave, considering that only 15.6% of the studied patients with seizures observed during this period had an epileptic preexisting condition compared to 37.5% of children observed during the Alpha/Delta variants spread.

As for pathogenesis of neurological involvement associated with SARS-CoV-2 infection, multiple mechanisms have been proposed, such as direct virus invasion, hyper-inflammatory reactions, associated multi-systemic failure, prothrombotic states and immune-mediated processes [29,30,31]. However, a causal relationship between SARS-CoV-2 infection and neurological manifestations is still unclear. In MIS-C patients, systemic inflammation and activation of cytokines has been considered as one of the key processes of post-infectious neurological involvement [32]. A role of inflammation in COVID-19 patients with neurological symptoms has also been reported [19]. We found a more pronounced inflammation (in terms of altered CRP) in patients with seizures compared to those without. Unfortunately, other inflammatory parameters such as ferritin or interleukin 6 were available in only few cases. A direct pathogenic role of the virus has been also hypothesized. Nevertheless, in a recent systematic review of patients with SARS-CoV-2-related neurological symptoms, the viral RNA has been detected in the CSF only in a few cases [33]. Unfortunately, we did not perform lumbar puncture in our patients because of the mild clinical course of the studied patients. Another possible hypothesis of neurological involvement in SARS-CoV-2 patients is a dissemination of the virus by the enteric nervous system, similar to what happens with Rotavirus gastroenteritis [34]. It is well known that Rotavirus can cause seizures [34]. On the other hand, the presentation of SARS-CoV-2 in children is characterized by gastrointestinal involvement with mild watery diarrhea [35]. In experimental models, it has been reported that Rotavirus induces a sequence of events leading to diarrhea, including early electrogenic chloride secretion via its nonstructural protein 4 (NSP4) enterotoxin followed by cytotoxic damage [36]. The same NSP4 has been suggested to be responsible for Rotavirus-associated seizures [37]. Very recently, it has been demonstrated that SARS-CoV-2 induces anion secretion in a model of human intestinal cells [38]. In this study, the spike protein, which is essential for SARS-CoV-2 binding and internalization, seemed to exhibit the typical features of an enterotoxin, similar to those of NSP4 in models of Rotavirus-induced diarrhea. Based on these in vitro data, one could speculate that the spike protein might be involved in a similar mechanism of neuroinvasion.

With regard to neuroimaging and EEG findings, they have been described only in few children with COVID-19 and seizures [16,18]. While in adults with COVID-19 and encephalopathy infarcts and abnormal cerebrovascular perfusion have been demonstrated, neuroimaging seems to be normal in most pediatric cases [16]. In our cohort, all performed brain CTs were unremarkable for new-onset findings. Despite brain CT not being the adequate technique to document a putative viral neurotropism and a direct neuroinvasion, it allows to rule out structural or acute cerebrovascular events as the cause of the seizures. Other diagnostic tools, namely brain MRI, positron-emission tomography and viral RNA search on the CSF, would have been more appropriate [39], but were not clinically indicated in our patients. EEG showed epileptiform abnormalities in 13% of the studied patients. None of them had a previous diagnosis of epilepsy. A comparison with the incidence of epilepsy in the general pediatric population was beyond the aims of our study; therefore, a role of SARS-CoV-2 infection in arousing the first epileptic manifestations cannot be affirmed based on our data. Further studies aimed at a more detailed neurophysiological characterization of COVID-19 children are desirable.

Our study has several limitations. First, our analysis was retrospective. However, all SARS-CoV-2-positive patients with seizures and complete medical records were included. Considering that our center is the largest pediatric hospital of Southern Italy, our sample may be considered a reliable surrogate of the SARS-CoV-2-infected pediatric population. Secondly, we did not perform a direct detection of the variant strains, but the attribution of patients to the Alpha/Delta or Omicron groups was based on epidemiological data only. Nevertheless, given the careful national monitoring of SARS-CoV-2 circulation, this methodology likely represents a sufficiently reliable approximation.

In conclusion, our findings suggest that although COVID-19 may potentially trigger seizures, they are generally not severe and do not require intensive care admission, similar to other common pediatric viruses. A small subgroup of these patients may present abnormal EEG and require antiepileptic treatment. The Omicron variant seems to be related to a higher risk of seizures compared to previous SARS-CoV-2 variants. Understanding the association between seizures and SARS-CoV-2 and defining their features may improve the clinical management of these patients. Studies with prolonged follow-up are required to better understand the incidence and prognosis of COVID-19-related seizures.

## Figures and Tables

**Table 1:** Baseline characteristics of patients with SARS-CoV-2 infection with and without seizures.

Variable	Patients with Seizures	Patients without Seizures	*p*
N	93	411	
Males ^+^	49 (52.7)	234 (56.9)	Ns
Age at admission (years) *	3.9 ± 3.7	2.5 ± 4.3	<0.0001
Underlying disease ^+^:			
- Any disease	33 (35.5)	67 (16.3)	<0.00001
- Congenital heart disease	1 (3)	12 (17.9)	0.04
- Chronic respiratory disease	1 (3)	6 (9)	Ns
- Congenital or chromosomal abnormalities	5 (15.2)	13 (19.4)	Ns
- Chronic renal disease	0	21 (31.3)	0.0003
- Epilepsy	18 (54.5)	6 (9)	<0.0001
- Autism spectrum disorder	6 (18.2)	7 (10.4)	Ns
- Others	2 (6.1)	2 (3)	Ns
Length of hospital stay (days) *	3.3 ± 2.5	2.7 ± 2.9	0.0003
SARS-CoV-2-related symptoms ^+^:			
- Fever	63 (67.7)	252 (61.3)	Ns
- Pharyngitis/pharyngodinia	11 (11.8)	15 (3.6)	0.003
- Cough	6 (6.4)	36 (8.8)	Ns
- Dyspnea	4 (4.3)	21 (5.1)	Ns
- Diarrhea	7 (7.5)	57 (13.9)	Ns
- Vomiting	11 (11.8)	48 (11.7)	Ns
- Others	5 (5.4)	13 (3.2)	Ns
Laboratory results:			
- WBC (count/µL) ^§^	7690 (5800–11,020)	7900 (6210–10,860)	Ns
- Platelets (count/µL) (×1000) ^§^	278 (222–339)	324 (272–419)	<0.0001
- CRP (mg/L) (n.v. < 5) ^§^	3.34 (0.73–9.56)	1.43 (0.63–4.74)	0.007
- Patients with altered CRP ^+^	41 (44.1)	98 (23.8)	0.004
- CPK (U/L) (n.v. < 145) ^§^	124 (81–168)	109 (78–151)	Ns

^+^ Number (percentage); * Mean and standard deviation; ^§^ Median (interquartile range); WBC = white blood count; CRP = C-reactive protein; CPK = creatine phosphokinase; n.v. = normal value.

**Table 2:** Comparison between patients with seizures observed before (Group 1) and after (Group 2) January 2022.

Variable	Group 1	Group 2	*p*
N	16	77	
Males ^+^	5 (31.3%)	44 (57.1%)	Ns
Age at admission (years) *	5.8 ± 4.8	3.4 ± 3.3	0.02
Underlying disease ^+^:			
- Any disease	9 (56.3)	24 (31.2)	Ns
- Congenital heart disease	1 (6.3)	0	Ns
- Chronic respiratory disease	1 (6.3)	0	Ns
- Congenital or chromosomal abnormalities	0	5 (6.5)	Ns
- Chronic renal disease	0	0	-
- Epilepsy	6 (37.5)	12 (15.6)	0.04
- Autism spectrum disorder	1 (6.3)	5 (6.5)	Ns
- Others	0	2 (2.6)	Ns
Length of hospital stay (days) *	3.3 ± 1.7	3.3 ± 2.6	Ns
Access to ED because of seizures ^+^	14 (87.5)	63 (81.8)	Ns
Febrile seizures ^+^	5 (31.3)	54 (70.1)	0.003
Type of seizure ^+^:			
- Partial	1 (6.2)	1 (1.3)	Ns
- Generalized	15 (93.8)	76 (98.7)	
Laboratory results:			
- WBC (count/µL) ^§^	7660 (5615–9855)	7740 (5800–11,460)	Ns
- Platelets (count/µL) (×1000) ^§^	281 (240–343)	278 (218–337)	Ns
- CRP (mg/L) (n.v. < 5) ^§^	0.73 (0.36–4.98)	3.5 (1–10.17)	0.02
- Patients with altered CRP^+^	4 (25)	37 (40.2)	Ns
- CPK (U/L) (n.v. < 145) ^§^	91.5 (77.5–117)	137 (88–191)	0.01
- Patients with altered CPK^+^	1 (6.2%)	5 (6.5%)	Ns

^+^ Number (percentage); * Mean and standard deviation; ^§^ Median (interquartile range); ED: emergency department; WBC = white blood count; CRP = C-reactive protein; CPK = creatine phosphokinase; n.v. = normal value.

## Data Availability

Not applicable.
